# Assessment of Surface Water Quality in the Krynka River Basin Using Fluorescence Spectroscopy Methods

**DOI:** 10.3390/plants14132014

**Published:** 2025-07-01

**Authors:** Sergey Chufitskiy, Sergey Romanchuk, Besarion Meskhi, Anastasiya Olshevskaya, Victoria Shevchenko, Mary Odabashyan, Svetlana Teplyakova, Anna Vershinina, Dmitry Savenkov

**Affiliations:** 1 Faculty of Biology, Donetsk State University, 24 Universitetskaya St., Donetsk 83001, Russia; s.romanchuk@donnu.ru; 2 Agribusiness Faculty, Don State Technical University, Rostov-on-Don 344000, Russia; spu-02@donstu.ru (B.M.); olshevskaya.av@gs.donstu.ru (A.O.); modabashyan@donstu.ru (M.O.); steplyakova@donstu.ru (S.T.); avershinina@donstu.ru (A.V.); savenkov-dstu@yandex.ru (D.S.)

**Keywords:** biomonitoring, chlorophyll fluorescence, phytoplankton, photopigments, Krynka River

## Abstract

This study presents a biomonitoring study of surface waters in the Krynka River basin, encompassing three major regional reservoirs: Khanzhenkovskoe, Olkhovskoe, and Zuyevskoe. These water bodies face significant anthropogenic pressure from mining effluents, industrial discharges, and domestic wastewater. Key pollutants identified are surfactants (SAAs), sulfates, phenols, chlorides, and manganese, with chemical oxygen demand (COD) exceeding regulatory limits. The research was conducted in September 2024. Based on the Specific Combinatorial Water Pollution Index, surface waters in the studied objects can be characterized as slightly polluted. To assess the negative impact of the identified pollutants on hydrobionts, the species composition of phytoplankton of the studied water bodies was analyzed. In the Olkhovskoe Reservoir and Olkhovaya River, cyanobacterial blooms (*Oscillatoria agardhii* G.) were observed, altering biodiversity in the Krynka River and Zuyevskoe Reservoir. Phytoplankton genera *Synedra*, *Amphiprora*, and *Navicula*—established bioindicators of aquatic ecosystem health—were dominant in Khanzhenkovskoe Reservoir, signaling nutrient enrichment and organic pollution. Changes in the species composition and structure of phytoplankton in the Krynka River, its tributaries and reservoirs, indicate a change in the level of saprobic water bodies from β to α-mesosaprobic, which indicates both the general level of surface water pollution and the accumulation of pollutants along the course of the river. The paper presents the results of fluorimetric analysis of photosynthetic activity of natural phytoplankton cells and demonstrates the possibility of using fluorescence induction curves for regular monitoring measurements. Fluorescence parameters indicate a general deterioration of photosynthetic activity of natural phytoplankton. The growth of *Oscillatoria agardhii* in the waters of the Olkhovskoe Reservoir and of green microalgae in the Zuevskoe Reservoir led to an increase in the fluorescence quantum yield (F_v_/F_m_) and the total photosynthetic activity index (PI), which makes it possible to use these parameters as indicator parameters reflecting the intensity of “blooming” of various phytoplankton species.

## 1. Introduction

Studies on assessment of surface water conditions in the Donets Ridge area are scattered and lack systematicity. As a rule, separate sections of river channels or specific water bodies are considered without taking into account the connection with other water bodies [1,2,3]. At the same time, there is no further assessment of the state of the general water basin of river systems. Most of such studies were conducted more than 10 years ago [1,2,3], and at present, insufficient attention is paid to the problems of the state of water resources in the region. At the same time, the state of surface waters in most studies was assessed as polluted [1,2,3,4]. Significant excess concentrations of nitrites (up to 12.8 mg L^−1^) and nitrates (up to 2180 mg L^−1^) of heavy metals were observed in the surface waters of the region: manganese—up to 7.5 mg L^−1^, lead—up to 0.25 mg L^−1^, arsenic—up to 1.2 mg L^−1^, zinc—up to 25 mg L^−1^ [1]. The nature of surface water pollution is similar for most water bodies of the region. Kalmius and Mius Rivers [4], which are part of the Azov Sea basin, are also subjected to intensive pollution. River pollution is one of the causes of changes in the state of the coastal water area of the Azov Sea [5,6,7], which indicates the scale of negative processes caused by anthropogenic load.

Along with the identification of the main pollutants entering surface waters with mine and industrial wastewater, an important aspect of assessing the state of the aquatic environment is biodiagnostics and phytomonitoring [8,9,10,11,12]. According to long-term data [11], the technogenic transformation of the region leads to a change in the state of indicator plant species and a change in the species composition of ecosystems. In earlier studies for the Olkhovaya River [4] it was shown that microalgae of the genera *Coelastrum*, *Tetraedron*, *Monoraphidium*, *Closteriopsis*, *Ulotrix*, *Amphora*, *Pinnularia*, *Merismopedia*; they are sensitive to cadmium contamination in the concentration range from 7 to 10 µg L^−1^, as well as high concentrations of sulfate ions (600–700 mg L^−1^), which was caused by the ingress of mine wastewater, while representatives of the genera *Nitzshia*, *Chlorella* and *Scenedesmus* are somewhat resistant to this type of pollution. When assessing the state of aquatic ecosystems, natural phytoplankton is a good bioindicator [13]. Phytoplankton communities act as indicator organisms in assessing the state of river systems [14], lakes [15] and seas [16]. Studies in this area are aimed both at searching for individual indicator species and at analyzing entire communities [17], assessing the pigment composition of surface waters [15]. The possibilities of using fluorimetric assessment of the photosynthetic activity of hydrobionts significantly expand the possibilities of express assessment of the state of the environment, and also allow identifying sources of pollution at the early stages of pollutants entering surface waters [18,19,20]. A promising direction for monitoring measurements is the registration and analysis of fluorescence induction curves, also called OJIP curves [21]. These curves reflect the processes of electron transport and excitation light energy transfer in photosystem II and its antenna complexes [21]. Methods of fluorimetric estimation of photosynthetic activity of phytoplankton are characterized by fast and informative measurements, do not require preliminary extraction of pigments and are suitable for routine monitoring measurements [18,19,20,22,23]. However, significant variability of phytoplankton species diversity and peculiarities of water bodies under study (water turbidity, current velocity, etc.) can influence the measurement results. In addition, the results of measurements can be affected by bio-optical processes of fluorescence absorption and quenching caused by both the content of dissolved organic substances and biotic factors [24]. This creates the need to develop optimal algorithms for measurements of large water bodies, river and sea basins [18,19,20,22,23].

To study the condition of the Krynka River and its tributaries, a section was selected that holds the greatest interest from an environmental, strategic, and social point of view. The river basin includes three large drinking water reservoirs: Khanzhenkovskoe, Olkhovskoe, and Zuyevskoe, which supply most of the region with drinking water. In the context of intense anthropogenic pressure on the river basin, the problem of monitoring the quality of surface waters and reservoirs is relevant and requires detailed study. The Zuevsky Regional Landscape Park is located near the Zuyevskoe Reservoir, and individual parts of the reservoir shorelines are recreation areas for the local population. For the Krynka River, there are only scattered data for various reporting periods, which typically do not reflect the full extent of pollution. The Olkhovaya River, a right-bank tributary of the Krynka River, is subject to intense pollution by sludge discharges and mine waters [4]. Most of the sludge and other pollutants accumulate in the Olkhovskoe Reservoir. Additionally, the pollution of the Olkhovaya River is fed by its left tributary, the Olkhovka River, which is also exposed to mine waters. The Khazhenkovskoye Reservoir is less exposed to anthropogenic pollution in the steppe than the Olkhovskoe Reservoir. However, as a result of active water use throughout the year, there is a significant decrease in reservoir water levels, which leads to the death of part of the biota and deterioration in the quality of surface waters. The Zuyevskoe Reservoir is most exposed to anthropogenic pollution. This reservoir is not a drinking water reservoir, but is part of the Krynka River basin, which flows into the Mius River, which flows into the Azov Sea [7]. The Zuyevskoe Reservoir is exposed to intense mine, industrial and domestic wastewater. Given the strategic importance of these water bodies and the degree of pollution of surface waters, there is a need to monitor the state of the Krynka River basin.

The purpose of the study was to investigate the condition of surface waters of the Krynka River taking into account the Olkhovskoe, Zuevskoye, and Khanzhenkovskoe Reservoirs and the left tributary—the Olkhovaya River. The study presents the research results for September 2024.

## 2. Materials and Methods

### 2.1. Monitoring Points and Research Objects

**Figure 1:**
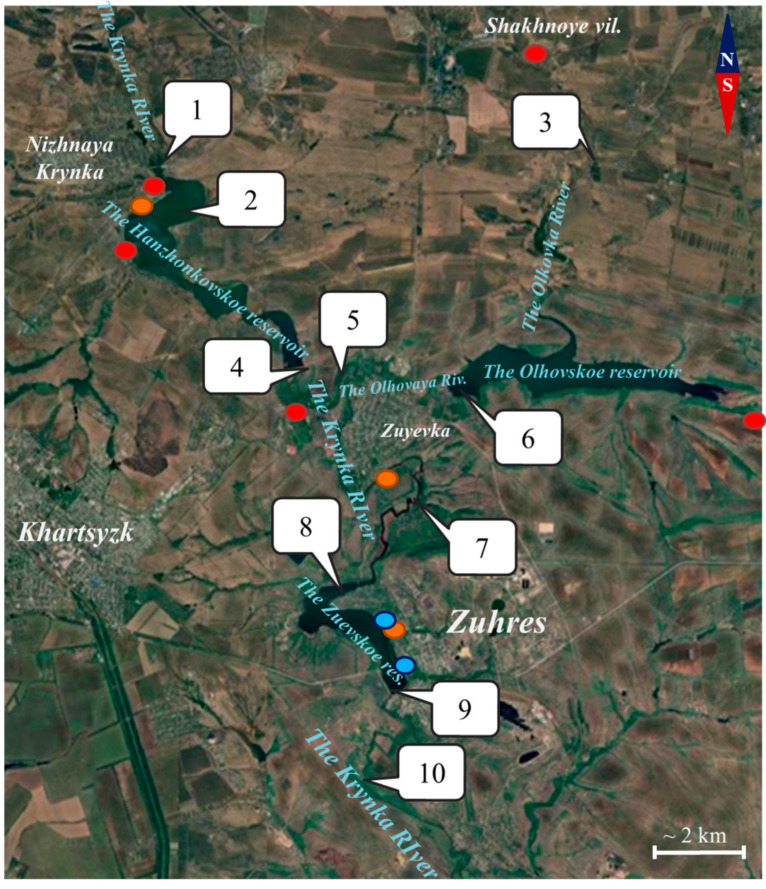
Location of monitoring points for the rivers Krynka, Olkhovka, Olkhovaya and reservoirs with suspected contamination sites: red circle—mine waters, orange circle—domestic wastewater, blue circle—industrial wastewater.

At the time of sampling, significant shallowing was occurring for the Khanzhenkovskoe reservoir. To monitor the condition of this part of the Krynka River basin, 10 monitoring points were selected, which are shown in Figure 1.

Constructed on the Krynka River between the settlements of Nyzhnya Krynka and Zuyevka, Khanzhenkivske Reservoir spans 4.8 km^2^, with a catchment area of 780 km^2^, water storage capacity of 19.4 million m^3^, and a length of 7.5 km. The first monitoring point (48°07′26.2″ N 38°10′32.8″ E) is located in the bed of the Krynka River, before the river flows into the reservoir (point 1). On the territory of the reservoir monitoring points were established as follows: 2 (48°06′18.9″ N 38°11′07.5″ E) in the upper reaches of the reservoir and 4 (48°04′19.7″ N 38°13′24.6″ E) in the lower reaches, on the dam near the spillway. Water samples from the upper reaches of the reservoir had higher turbidity, as well as a high content of zooplankton, in particular daphnia and cyclops crustaceans.

Olkhovskoe Reservoir is formed by the Olkhova River, a left tributary of the Krynka River, Olkhovske Reservoir spans 3.7 km^2^ and extends 6 km in length. Over 20 coal-mining enterprises discharge wastewater into the hydrological network of this drinking water reservoir. The Olkhovka River itself is subjected to intense anthropogenic pressure [4].

The Olkhovka River passes through several mining settlements, where it is exposed to mine wastewater, and flows into the reservoir in its northern part. Monitoring point 3 (48°06′37.2″ N 38°18′25.6″ E) was located in the Olkhovka River bed near the settlement of Olkhovka. Water samples from the Olkhovskoe Reservoir were taken in the area of the dam, from the side of the settlement of Zuyevka (point 6 (48°04′01.4″ N 38°16′18.9″ E)).

On the territory of the settlement Zuyevka there is a confluence of the Olkhovaya and Krynka rivers, after these rivers exit the reservoirs. Point 5 (48°04′13.7″ N 38°14′14.5″ E) was located in the channel of the Olkhovaya river, before flowing into the Krynka river, point 7 (48°03′00.4″ N 38°14′54.4″ E)—outside the territory of the settlement Zuyevka, after the confluence of the Krynka and Olkhovaya rivers. The Zuyevskoe Reservoir was formed by the Krynka River and is one of the largest industrial reservoirs in the region. The reservoir has a surface area of 2.5 km^2^, a catchment area of 1327 km^2^, a water storage capacity of 5.9 million m^3^, and a length of approximately 4.5 km. Unlike Olkhovskoe and Khanzhenkovskoe, this reservoir is not a drinking water reservoir and meets the technological needs of the Zuyevskaya thermal power plant. In addition, this reservoir allows regulating the hydro regime of the Krynka River. In addition to the man-made load from the power plant, this reservoir is subject to the discharge of mine waters, as well as rainwater, domestic and industrial wastewater from the city of Zuhres, located on the left (eastern) bank of the reservoir (Figure 1). To assess the condition of the reservoir, two monitoring points were selected: point 8 (48°01′37.8″ N 38°13′36.8″ E)—the upper reaches of the reservoir, the coast of the village of Vodobud, and point 9 (48°00′34.9″ N 38°15′20.4″ E)—the reservoir dam, the city of Zuhres. The condition of surface waters in the Krynka River after the reservoirs was assessed by monitoring point 10 (47°58′41.0″ N 38°16′36.3″ E), on the territory of the village of Shakhtnoye.

Water samples were taken from the surface coastal layer of water bodies in the morning and afternoon over one day. Three liters of water were taken at each monitoring point to perform the analysis of samples’ physical and chemical composition, assessment of species and pigment composition of phytoplankton, and fluorimetric measurements.

### 2.2. Determination of the Physicochemical Composition of Water Samples

The physicochemical composition of water samples was analyzed using Hach cuvette tests (LCK 014, LCK 304, LCK 311, LCK 320, LCK 329, LCK 332, LCK 338, LCK 340, LCK 341, LCK 345, LCK 349, LCK 353, LCK 394). Measurements were performed on a Hach DR 3900 spectrophotometer (Loveland, CO, USA). Results were compared to maximum permissible concentrations (MPC) per SanPiN 2.1.4.1074-01 [25], which is a normative document regulating the values of permissible concentrations of most chemical substances in natural surface waters.

Water quality was assessed by determining the water pollution class based on the Specific Combinatorial Water Pollution Index (SCWPI) and generalized assessment scores (S_i_) for each component. SCWPI is calculated using the following formula:

$$SCWPI=\sum_{i=0}^{n}S_{i}=\sum_{i=0}^{n}\left(\frac{C_{i}}{{MPC}_{i}}\cdot \frac{N_{{MPC}_{i}}}{N_{i}}\right),$$

where C_i_—concentration of i-th substance in water sample; ${MPC}_{i}$—maximum permissible concentration of i-th substance in a water sample; $N_{{MPC}_{i}}$—number of exceedances of the MPC value for the i-th substance; $N_{i}$—total number of measurements of the i-th substance content in the medium.

### 2.3. Water Sampling and Determination of Phytoplankton Species Composition

Sampling was carried out in the morning and afternoon in plastic or glass containers with a volume of 1.5 dm^3^. Samples were collected in October 2023. To determine the species composition of phytoplankton in laboratory conditions, samples were concentrated by filtration through MFAS-OS-4 membrane filters from Vladipor (Vladimir, Russia) with a pore diameter of 0.6 μm. The species composition of the samples was determined using a light microscope with a magnification of 600. Cell counting was performed using a Goryaev chamber («MicMed», Saint-Petersburg, Russia), according to the following formula:

$$N=k\cdot n\cdot \left(\frac{A}{a}\right)\cdot v\cdot \left(\frac{1000}{V}\right),$$

where N—the number of organisms in a liter of water in the studied reservoir; k—coefficient showing how many times the volume of the counting chamber is smaller 1 cm^3^; n—number of cells found in the scanned squares of the camera; A—total number of squares in the counting chamber; a—number of squares in which algae were counted; v—volume of sample concentrate (cm^3^); V—initial volume of the sample taken (cm^3^).

When determining the species affiliation of microalgae, classification schemes were used according to [26].

### 2.4. Spectrophotometric Determination of Photopigment Content

The content of chlorophyll and other photopigments was determined according to the expressions proposed by S.W. Jeffrey and G.F. Humphrey [27,28]. Pigments were extracted using a 90% acetone solution. When estimating chlorophyll a content, a number of pigments may be present in the extract that interfere with the analysis: pheophytin a, pheophorbide a, and chlorophyllide a. Concentrations of chlorophyllide are generally disproportionately small compared to those of photopigment, so no correction for their presence was made. To determine the contribution of pheophytin and pheophorbide to chlorophyll content, the extract under study was acidified with hydrochloric acid at a concentration of 3–5 mmol/L. Thus, chlorophyll a concentrations without pheophytin and pheophorbide were obtained after acidification. To assess the diversity of the algocenosis by pigment composition, the Margalef pigment index [29] is used, which is defined as the ratio of the optical densities of the extract at 430 and 664 nm.

### 2.5. Methodology of Fluorimetric Research

Fluorimetric analysis of water samples was carried out using an FS-2 fluorimeter. During the study, the total chlorophyll content was measured, and fluorescence induction curves were recorded. Induction curves are also called OJIP curves, which correspond to the values of the local minimum of the curve (denoted as O) and three subsequent peaks—J, I and P, where P corresponds to the maximum value of fluorescence intensity. The curves were analyzed using the PyPhotoSyn program [30], determining and interpreting the OJIP test parameters as proposed in [31,32,33,34]. Main parameters are given in Table 1.

Before recording fluorescence induction curves, water samples were placed in a darkened place for 20 min for dark adaptation. No temperature control was performed; all measurements were performed at room temperature. For a single measurement, 3 mL of water was taken, and at least 10 measurements were performed for each sample.

The chlorophyll a content in water samples was determined by the F_0_ level using the fluorimetric method. For correct interpretation of F_0_ level and chlorophyll concentrations in water samples, the fluorimeter was calibrated using chlorophyll extracts from laboratory culture of green microalgae *Chlorella vulgaris*. For this purpose, algal cells were concentrated on membrane filters, and extraction of photopigment with acetone solution was carried out. The obtained extract was separated into five concentrations (0.05, 0.1, 0.5, 1, 2, and 2.5 μg/L), and the F_0_ level was measured. A calibration curve was constructed from the obtained data by linear approximation.

### 2.6. Data Analysis

Reliability of differences in mean values for all obtained results was determined using non-parametric statistics methods—Wilcoxon W-criterion—and the degree of correlation between samples was assessed using Spearman’s coefficient.

## 3. Results

### 3.1. Physicochemical Composition of Water Samples

The results of the measurements are presented in Table 2. Values in bold indicate exceedances of maximum permissible concentrations; the “–” sign means that no measurements were taken for the samples. The table shows only the measurement results for compounds whose concentrations exceeded the maximum permissible limits.

All water samples were characterized by excess sulfate, chloride, and phenol content. High sulfate and chloride concentrations in the Krynka River and its tributaries are due to the inflow of large volumes of mine water. These excesses are also constant, regardless of seasonal changes. Monitoring points 1 and 10 recorded excess maximum permissible concentrations (MPC) for manganese. Exceeding permissible levels of phenol and chemical oxygen demand (COD) indicates intense pollution of the Krynka River bed, its tributaries, and reservoirs. Low biogenic content in water samples indicates that phytoplankton growth and reproduction are limited by phosphorus concentration.

According to the data obtained, the water in the Krynka River bed can be characterized as slightly polluted (2nd pollution class) by SCWPI.

### 3.2. Species Composition of Phytoplankton in the Krynka River Basin

A total of 39 main phytoplankton species were identified. The richest species composition was observed in monitoring points of Khanzhenkovskoe and Zuyevskoe reservoirs. In the upper reaches of Khanzhenkovskoe Reservoir, *Amphiphora palugosa* W.Sm. dominated, while this species was nearly absent in the lower reaches, where representatives of the genus *Fragilaria* appeared. In Zuyevskoe Reservoir, green algae of the genera *Chlorella*, *Dictyosphaerium*, *Monoraphidium*, and *Scenedesmus dominated*.

Species found in almost all monitoring points included *Chlorella vulgaris* Beij., *Oscillatoria agardnii*, *Synedra acus* K., *Synedra ulna* E., and representatives of the genera *Navicula* and *Ankistrodesmus*. Dominant species in specific monitoring points included *Amphiphora palugosa*, *Chlorella vulgaris*, *Monoraphidium contortum* Thur., *Oscillatoria agardnii*, *Scenedesmus quadricauda* B., *Scenedesmus acuminatus* Chodat, *Synedra acus*, and *Synedra ulna*. Rarely encountered species included *Actinastrum hantzschii* Lagerh., *Bacillaria paradoxa* J.F.Gmelin, *Chaetoceros muelleri* L., *Crucigenia tetrapedia* K., *Monomorphina pyrum* M., and representatives of the genera *Cymbella* and *Trachelomonas*.

The division Bacillariophyta was the most diverse, comprising four classes, ten orders, eleven families, and thirteen species (Table 3). Chlorophyta included three orders, five families, thirteen genera, and nineteen species. Cyanobacteria comprised four orders, four families, four genera, and four species. Euglenozoa was represented by several genera.

In the Krynka River (Point 1), Bacillariophyta accounted for 96% of total abundance, with ~90% belonging to the genus *Synedra*. Euglenophytes constituted 3% (1.3% *Phacus* spp., 1.2% *Euglena* spp.). Green algae were represented by *Chlorella*.

In the upper Khanzhenkovskoe Reservoir (Point 2), diatoms comprised 82.6%, green algae 13.1%, and cyanobacteria 4.3%. Dominance shifted from *Synedra* to *Amphiphora palugosa* (50% of diatoms). *Synedra acus* and *Synedra ulna* accounted for 13%, with *Cyclotella* and *Navicula* also prevalent. Green algae included *Ankistrodesmus longissimus* Wille and *Chlorella vulgaris*. Cyanobacteria were dominated by *Oscillatoria agardnii* and *Oscillatoria limosa* C.Agardh ex Gomont. In the lower reservoir (point 4), diatoms decreased to 52%, while green and cyanobacteria increased to 33.6% and 14.5%, respectively. *Amphiphora palugosa* disappeared, with *Synedra* (20.5%), *Cyclotella* (~6%), *Navicula* (~6%), and *Fragilaria* (9.5%) dominating. Green algae remained dominated by *Ankistrodesmus longissimus* (18%) and *Chlorella vulgaris* (5.7%), while *Oscillatoria agardnii* increased to 11%.

In the Olkhovka River (point 3), phytoplankton abundance was minimal (<2 million cells L^−1^). Diatoms dominated (87%), with *Synedra* (36%) and *Tabellaria* (33%). *Tabellaria fenestrate* L. was dominant. Chlorophyta (7%) included only *Scenedesmus quadricauda*. Cyanobacteria and Euglenozoa each accounted for ≤2.6%. Blue-green algae included members of the genus *Oscillatoria*, and euglenoids included members of the genera *Euglena*, *Monomorphina*, *Phacus*, and *Trachelomonas*.

In the waters of Olkhovskoe Reservoir (point 6), cyanobacteria dominated (83%). The division Chlorophyta accounted for 11%, and Bacillariophyta for 5.2%. This distribution is uncharacteristic for the studied part of the Krynka River basin. The dominance of cyanobacteria persisted in the Olkhovaya River (point 5). In the reservoir, the species *Oscillatoria agardnii* proliferated intensively, constituting 80% of the total phytoplankton cell abundance. In the Olkhovaya River, this species also dominated, accounting for 61%. In the reservoir, green algae were represented by the genera *Ankistrodesmus*, *Chlorella*, *Dictyosphaerium*, and *Scenedesmus*. The species *Dictyosphaerium pulchellum* H.C.Wood accounted for approximately 3.8% of the abundance, *Chlorella vulgaris* for 2.6%, and *Ankistrodesmus longissimus* for 2.4%. In the Olkhovaya River, green algae were practically absent. In the reservoir waters, diatoms constituted about 5% of the total abundance. In Olkhovskoe Reservoir, the species *Chaetoceros muelleri* was found, which was not detected at any other monitoring point. Most diatoms consisted of the species *Synedra acus* and *Synedra ulna*. In the Olkhovaya River, in addition to the genus *Synedra*, species of the genus *Cyclotella* proliferated.

The confluence of the Olkhovaya and Krynka Rivers (Point 7) led to changes in the species composition in the Krynka River and the dominance of cyanobacteria, which accounted for 73% of the abundance. The dominance of cyanobacteria was due to the high abundance of *Oscillatoria agardnii* (71% of the total abundance). The species *Oscillatoria planctonica* Vangoor was also found in these samples. The division Bacillariophyta accounted for 23% of the abundance and was represented mainly by species of the genera *Amphiphora*, *Cyclotella*, *Navicula*, *Nitzschia*, and *Synedra*. The species composition of diatoms at this monitoring point is similar to that in the lower reaches of Khanzhenkovskoe Reservoir. Green algae were represented only by the species *Ankistrodesmus longissimus* and *Chlorella vulgaris* and the genus *Oocystis*. Since green algae were absent in the Olkhovaya River channel, these species likely originated from Khanzhenkovskoe Reservoir.

In Zuyevskoe Reservoir, a redistribution of dominant phytoplankton species occurred. Compared to the Krynka River channel (Point 7), the overall cyanobacteria content decreased, and green algae increased. In the upper reaches of the reservoir (Point 8), the division Chlorophyta accounted for 73% of the total phytoplankton abundance. Algae of the genera *Chlorella* (17.3%) and *Scenedesmus* (16.5%) predominated, with the dominant species being *Chlorella vulgaris* and *Scenedesmus quadricauda*. Frequently encountered species also included *Monoraphidium* (10.8%), *Ankistrodesmus* (7.2%), and *Dictyosphaerium* (6.5%). The division Bacillariophyta accounted for 15.8% of the abundance, with the dominant species *Synedra acus* (6.5%). In addition to the genus *Synedra*, cells of the genus *Navicula* were found in the samples. The cyanobacteria abundance was primarily composed of oscillatorians and the species *Oscillatoria agardnii* (10.81%). In the lower reaches of Zuyevskoe Reservoir (Point 9), the proportion of cyanobacteria increased to 18%, of which 14% were oscillatorians. Representatives of the genera *Microcystis* and *Merismopedia* were also found. The composition structure of green algae remained unchanged, with an increase in the proportion of *Scenedesmus quadricauda* cells to 20.7%. In the lower reaches, proliferation of the genera *Kirchneriella* and *Crucigenia* was observed, particularly the species *Crucigenia fenestrata* Schmidle. The total proportion of the Chlorophyta division was 71%. The species composition structure of diatoms remained unchanged, with dominance by species of the genus *Synedra*.

In the Krynka River channel downstream of Zuyevskoe Reservoir (Point 10), an increase in the proportion of green algae to 87% and a decrease in cyanobacteria abundance to 1.4% were observed. The genus *Ankistrodesmus* was represented by cells of the species *Ankistrodesmus falcatus* Ralfs (6.3%), while the species *Ankistrodesmus longissimus* was practically absent. Dominance of the species *Chlorella vulgaris* (19.9%) and *Monoraphidium contortum* (22.3%) persisted. The genus *Scenedesmus* was predominantly represented by two species: *Scenedesmus quadricauda* (9.3%) and *Scenedesmus acuminatus* (12.3%). Cyanobacteria were represented by the species *Oscillatoria agardnii*. The abundance of diatoms was comparable to that in the lower reaches of Zuyevskoe Reservoir and amounted to 10.3%. The Bacillariophyta division was equally represented by the genera *Cyclotella*, *Melosira*, and *Synedra* (3% each). Euglenophytes were also more frequently encountered in this section of the channel, accounting for approximately 1.4% of the abundance.

The change in dominant species and individual taxa in different areas of the studied water bodies indicates the instability of the algocenosis structure. Total dominance of one algal species is characteristic of the upper reaches of the Khanjenkovskoe and Olkhovskoe Reservoirs. The growth of cyanobacteria *Oscillatoria agardnii* in the waters of the Olkhovskoe Reservoir and the Olkhovaya River is close to blooming. The majority of dominant species belong to β-mesosaprobic species: *Synedra acus*, *Oscillatoria agardnii*, and *Dictyosphaerium pulchellum*, the presence of which indicates a moderate degree of surface water pollution. These species are dominant in Khanzhenkovskoe and Olkhovskoe Reservoirs. At the confluence of the Krynka River with the Olkhovaya River, the dominant species are redistributed, and α-mesosaprobic species (e.g., *Chlorella vulgaris*) appear among the dominants in the waters of the Zuevskoe Reservoir, indicating increased pollution of the aquatic environment as well as a decrease in dissolved oxygen content. Chemical analysis of water samples revealed a light degree of pollution, but the change in phytoplankton structure indicates a more intense pollution of surface waters. Probably, the list of chemical pollutants (Table 2) does not include a number of other pollutants that have a negative impact on biota.

### 3.3. Spectrophotometric Determination of Photopigment Composition of Water Samples

The composition of photopigments was determined at monitoring points 1, 3, 4, 6, 7 and 10. These points were characterized by the predominance of chlorophyll a and c (Table 4). The presence of cyanobacteria was manifested in the presence of carotenoids of these algae.

The lowest total photopigment content was obtained for the Olkhovskoe Reservoir, the Olkhovaya River, and the Krynka River (point 7). At points 5 and 6, significant development of cyanobacteria and a very low content of diatoms and green algae, which dominated at the other monitoring points, were observed. Low photopigment content was also observed for the Olkhovka River. Changes in photopigment concentrations were also observed in the Khanzhenkovskoe Reservoir. Compared to the Krynka River (point 1), the chlorophyll a content increased in the lower reaches of the reservoir due to the growth of green algae.

Overall, the photopigment content was highest in the upper reaches of the Krynka River (Point 1) and the Khanzhenkovskoe Reservoir (Point 4). In the lower reaches of the Krynka River, as well as in the Zuyevskoe and Olkhovskoe reservoirs, the content of photopigments was lower by about 5 times. The decrease in the total content of pigments could have occurred due to the pollution of the Krynka River basin with phenols (Table 2).

The Margalef pigment indices for the studied monitoring points fluctuated within 1.9–2.5. Only at monitoring point 7 did the index increase to 3.3. According to the species composition, a change in the dominant species occurred at this point, which came from the left tributary of the Krynka River—the Olkhovaya River. The values of the pigment index in the range from 1 to 2 are usually characteristic for young algocenoses, and values from 3 to 5 are characteristic for aging communities, as well as for water bodies with unfavorable environmental conditions for phytoplankton growth [29].

**Figure 2:**
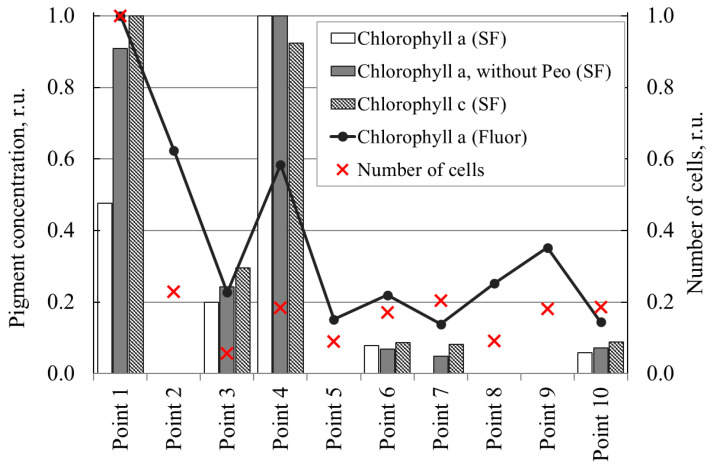
Concentrations of photopigments and the phytoplankton abundance in the studied monitoring points of the Krynka River basin: SF—photopigment concentration, measured by spectrophotometry; Fluor—photopigment concentration, measured by fluorometry.

The fluorimetric determination of chlorophyll content agrees with the results of spectrophotometry. Figure 2 shows the results of spectrophotometric (diagram columns, designated as “SF” in the legend) and fluorimetric (as a line, designated as “Fluor” in the legend) determination of the content of photosynthetic pigments in water samples. There are no chart columns for individual monitoring points because no measurements were taken for them. The change in the phytoplankton abundance is shown as individual points (marker ꓫ). All presented data are normalized to the maximum sample value.

Table 5 presents the values of the Pearson correlation coefficients between the main quantitative indicators of phytoplankton. Values in bold indicate high level of correlation between indicators.

Strong positive correlations are observed between the cell count and the photopigment content measured by fluorimetry, as well as between the spectrophotometry and fluorimetry methods. Medium and weak correlations are obtained between the cell count and the spectrophotometric method for determining photopigment concentrations.

### 3.4. Analysis of Photosynthetic Activity of Natural Phytoplankton of the Kalmius River

**Figure 3:**
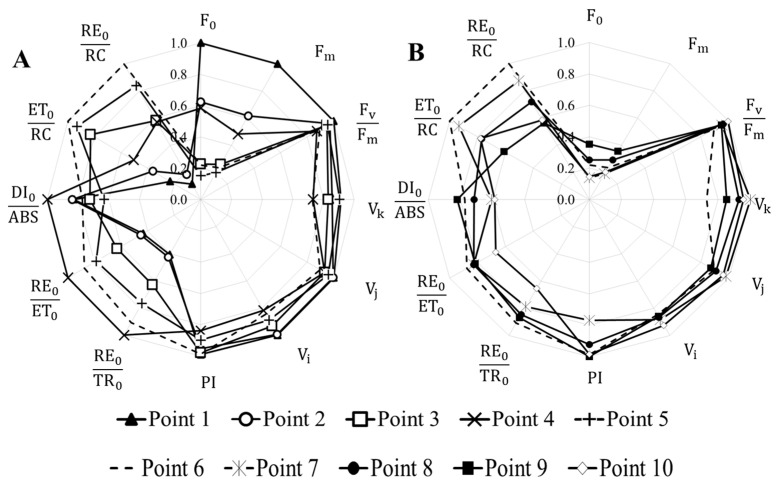
Parameters of the OJIP test of chlorophyll induction curves of phytoplankton in the Krynka River basin: (**A**). Results for monitoring points from 1 to 6; (**B**). Results for monitoring points from 6 to 10.

Among the general list of OJIP test parameters, 12 parameters were identified that varied for different monitoring points and reflected changes in the functional state of the photosynthetic apparatus of phytoplankton. Figure 3 shows the petal diagrams of the OJIP test parameters for which reliable differences were found between individual monitoring points. For a more convenient display, the monitoring points are divided into two diagrams (Figure 3A,B) relative to monitoring point 6.

Changes in photosynthetic activity of phytoplankton can be traced in relation to the confluence of the Krynka and Olkhovaya Rivers. Constant changes in the species composition and changes in dominant forms of microalgae caused significant changes in the distribution of fluorescence induction curve parameters (Figure 3A). For phytoplankton from the Zuevskoe Reservoir and the Krynka River channel after the Olkhovskoe Reservoir, the change in fluorescence indices became less significant (Figure 3B), which is due to the presence of expressed dominant forms of phytoplankton.

**Figure 4:**
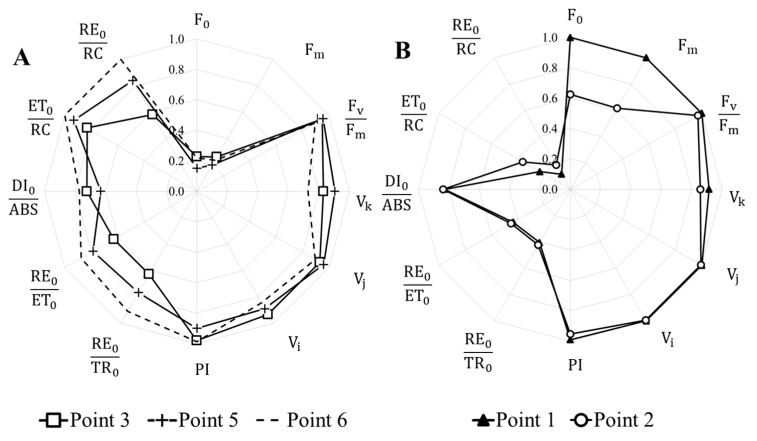
Parameters of the OJIP test of some monitoring points of the Krynka River basin: (**A**). Olkhovka and Olkhovaya Rivers and Olkhovskoe Reservoir; (**B**). Krynka River and Khanzhenkovskoe Reservoir.

In the Krynka River, before its inflow into the Khanzhenkovskoe Reservoir, the highest intensity of chlorophyll fluorescence was detected, which indicates a high content of phytoplankton in the samples and is consistent with other measurement results (Figure 2). In this area, the lowest values for the electron flow between photosystems II and I (ET_0_ RC^−1^ and RE_0_ RC^−1^) were noted for phytoplankton cells, as well as low rates of electron transfer between PS II acceptors (RE_0_ TR_0_^−1^ and ET_0_ TR_0_^−1^). Despite the change in dominant species between points 1 and 2, the fluorescence values for these samples were similar (Figure 4B). Changes occurred only in the intensity of maximum and minimum fluorescence.

According to the results of chemical analysis of samples (Table 2), at monitoring point 1 there was contamination with anionic surfactants, and concentrations of manganese and phenol exceeding the MPC were also present. The phenol content exceeded the maximum permissible concentration by 5 times. At point 1, over 90% of the total abundance was represented by the genus *Synedra*, while in the upper reservoir (point 2), the species *Amphiphora palugosa* accounted for 50% of the abundance, and the genus *Synedra* constituted approximately 13% of phytoplankton abundance. The third most abundant genus in these monitoring points was *Navicula*: 2% at point 1 and 11% at point 2. Thus, these diatom genera can be considered the primary contributors to the fluorescence signal. Species of the genera *Synedra* (particularly *Synedra ulna*) and *Navicula* (particularly *Navicula cryptocephala* Rabenh.) are classified as β-mesosaprobic [13,35,36,37,38]. Additionally, at the time of sampling, a significant decrease in the reservoir water level was observed, which also affected surface water quality and the state of hydrobionts.

Similar changes in the photosynthetic activity of phytoplankton were also characteristic of the Olkhovaya River basin (Figure 4A). In the Olkhovka and Olkhovaya Rivers and the Olkhovskoe Reservoir, a low content of photopigments was observed, while the photosynthetic activity of phytoplankton cells was high. High values of the general indicators of the efficiency of photosystems were noted—PI and F_v_ F_m_^−1^, as well as parameters of the efficiency of the functioning of the electron transport chain between photosystems I and II. In Olkhovskoe Reservoir and the channel of the Olkhovaya River, intensive proliferation of *Oscillatoria agardnii* occurred. The abundance of this species in the river was higher than in the reservoir, indicating its active growth. The high photosynthetic activity levels are most likely linked to the expansion of this species. Representatives of the genus *Synedra* were identified in all three monitoring points.

Phytoplankton from water samples of the Zuevskoye Reservoir and the Krynka River, after the confluence with the Olkhovaya River, was characterized by high rates of photosynthetic activity. However, in this area there was a constant redistribution of dominant species with their growth and subsequent replacement by the next dominants. Therefore, in these samples, high photosynthetic activity is due to the active process of reproduction of dominant forms of phytoplankton, which did not allow us to identify any negative impact on other phytoplankton species based on the total fluorescence signal.

## 4. Conclusions

Surface water in the studied monitoring points can be assessed as slightly polluted based on the analysis of the chemical composition of samples and SCWPI. The increased content of sulfates can be attributed to the peculiarities of the mineral composition of water bodies in the region, because in a number of other rivers [4,7,12] the increased content of these ions is also observed. Pollution with anionic surface substances and phenol can cause the death of a significant amount of biota.

The specific hydrological regime of the Krynka River, the presence of a cascade of interconnected reservoirs, and changes in the physicochemical composition of the water led to alterations in the phytoplankton species composition, with shifts in dominant species in the channel of the Krynka River. Based on the phytoplankton species composition, surface waters of the Krynka River basin in the study area can be classified as β-mesosaprobic with a gradual transition to α-mesosaprobic type in the Zuevskoye Reservoir. This indicates more intensive pollution of surface water than that detected on the basis of chemical analysis. Based on changes in phytoplankton species composition, surface waters can be attributed to the polluted or highly polluted type.

Algae of the genus *Synedra* and the species *Amphiphora palugosa*, identified in Khanzhenkovskoe Reservoir, can be considered indicator species, as changes in their photosynthetic activity are associated with contamination of surface waters by anionic surfactants and phenol. Under conditions of significant dominance of one microalgae species in Khanzhenkovskoe and Olkhovskoe Reservoirs, fluorescence induction curves can act as a strong tool for assessment of photosynthetic activity. The construction of relationships between chemical pollution and changes in the physiological state of cells of dominant phytoplankton is much easier in this case. In the presence of several dominant species, the total share of which in the algocenosis is the same, the comparison of changes in environmental factors and photosynthetic activity of algae becomes more complicated, which can be attributed to the disadvantages of the method. Based on the changes in the phytoplankton species composition, it can be assumed that the degree of saprobicity of the aquatic environment in the Zuevskoe Reservoir has increased; however, according to fluorimetry data, the photosynthetic activity of phytoplankton cells increased. This is primarily due to the intensive growth of green microalgae, the total fluorescence signal of which can offset the contribution of other species. Thus, the fluorimetry method is an informative and fast method for assessing photosynthetic activity of microalgae; however, when studying the state of natural communities, it is necessary to strictly take into account species diversity to avoid errors in the interpretation of the results obtained.

## Figures and Tables

**Table 1:** OJIP-test parameters [34].

F;_0_	Minimal fluorescence
F_m_	Maximum fluorescence
F_v_ F_m_^−1^	Maximum quantum fluorescence yield
V_j_	Variable fluorescence at J-phase, reflects the proportion of closed reaction centers in photosystem I
V_i_	Variable fluorescence at I-phase, reflects the ability of photosystem I acceptors to oxidize a plastoquinone pool
V_k_	Variable fluorescence at 0.3 ms
PI	Total photosynthetic activity performance index
RE_0_ ET_0_^−1^	Efficiency of electron transfer from plastoquinone to photosystem I acceptors
ET_0_ ABS^−1^	Quantum yield of electron transport between primary quinone and plastoquinone pool
RE_0_ ABS^−1^	Quantum yield of electron transport between primary quinone and to photosystem I acceptors
DI_0_ ABS^−1^	Quantum yield of energy dissipation in photosystem II antennae
RE_0_ RC^−1^	The flux of electrons transferred between primary quinone and to photosystem I acceptors
ET_0_ RC^−1^	The flux of electrons transferred between primary quinone and plastoquinone pool
TR_0_ RC^−1^	Maximum trapped exciton flux per active photosystem II
DI_0_ RC^−1^	The flux of dissipated energy

**Table 2:** Physicochemical composition of water samples from the rivers Krynka, Olkhovka and reservoirs.

Indicators, mg L^−1^	MPC	Point 1	Point 2	Point 3	Point 4	Point 5	Point 6	Point 7	Point 8	Point 9	Point 10
Anionic surfactants	0.5	**0.53**	0.34	0.22	0.39	0.11	0.10	0.26	0.20	0.37	0.26
Sulfates	500	**658**	**645**	**757**	**656**	**766**	**664**	**773**	**863**	**934**	**815**
Chlorides	350	**998**	**907**	**922**	**810**	**589**	**570**	**643**	**757**	**826**	**865**
Phenol	0.1	**0.56**	**0.17**	**0.18**	**0.17**	**0.11**	**0.11**	**0.12**	**0.16**	**0.16**	**0.23**
Ammonium	1.5	0.62	<0.1	<0.1	0.77	<0.1	<0.1	0.19	<0.1	<0.1	**1.87**
Chemical oxygen demand	15	**42.8**	**29.7**	13.4	**35.5**	**15.7**	**18.4**	**20.9**	**26.4**	**28.4**	**24.1**
Manganese	0.1	**0.20**	0.04	<0.02	0.03	0.02	<0.02	<0.02	<0.02	0.02	**0.21**

**Table 3:** Systematic structure of phytoplankton of the Krynka River.

Division	Class	Order	Family	Genus	Species	Identified to Genus
Bacillariophyta	4	10	11	15	13	10
Chlorophyta	2	3	5	13	19	5
Cyanobacteria	1	4	4	4	4	3
Euglenozoa	1	1	2	3	3	3
Total	8	18	22	35	39	21

**Table 4:** Content of photopigments in water samples from the Krynka, Olkhovka, Olkhovaya rivers and reservoirs.

Pigments, µg L^−1^	Chlorophyll a	Chlorophyll a (Without Pheophytin)	Pheophytin	Chlorophyll b	Chlorophyll c_1_ and c_2_	Cyanobacterial Carotenoids	Diatom Carotenoids	Pigment Index (D_430_/D_664_)
Point 1	22.79	53.25	52.20	0.00	244.56	13.54	33.86	2.26
Point 3	9.56	14.24	8.08	0.00	72.20	4.44	11.10	2.48
Point 4	47.82	58.62	18.83	0.00	225.78	21.91	54.77	1.92
Point 6	3.73	4.01	0.51	0.00	21.21	2.52	6.31	2.57
Point 7	0.00	2.83	6.79	0.00	19.91	1.79	4.47	3.33
Point 10	2.83	4.25	2.45	0.00	21.77	1.67	4.18	2.50

**Table 5:** Values of the correlation coefficients between the phytoplankton abundance and the content of photopigments in water samples from the Krynka, Olkhovka, Olkhovaya rivers and reservoirs.

	Chlorophyll a (Fluor)	Number	Chlorophyll a (SF)	Chlorophyll a, Without Pheo (SF)	Chlorophyll c (SF)
Chlorophyll a (Fluor)		**0.826**	0.660	**0.890**	**0.934**
Number	**0.826**		0.230	0.571	0.654
Chlorophyll a (SF)	0.660	0.230		**0.926**	**0.873**
Chlorophyll a, without Pheo (SF)	**0.890**	0.571	**0.926**		**0.992**
Chlorophyll c (SF)	**0.934**	0.654	**0.873**	**0.992**	

## Data Availability

The datasets presented in this article are not readily available because the data are part of an on-going study. Requests to access the datasets should be directed to: chufitsky@donnu.ru.

## Abbreviations

SAAs	surfactants
COD	chemical oxygen demand
MPC	maximum permissible concentrations
SCWPI	specific Combinatorial Water Pollution Index
